# Bottlenose dolphins can understand their partner's role in a cooperative task

**DOI:** 10.1098/rspb.2018.0948

**Published:** 2018-09-19

**Authors:** Kelly Jaakkola, Emily Guarino, Katy Donegan, Stephanie L. King

**Affiliations:** 1 Dolphin Research Center, 58901 Overseas Highway, Grassy Key, FL 33050, USA; 2 Centre for Evolutionary Biology, School of Biological Sciences, University of Western Australia, Crawley 6009, Australia

**Keywords:** cooperation, bottlenose dolphins, problem-solving, comparative cognition, synchrony, joint action

## Abstract

In recent decades, a number of studies have examined whether various non-human animals understand their partner's role in cooperative situations. Yet the relatively tolerant timing requirements of these tasks make it theoretically possible for animals to succeed by using simple behavioural strategies rather than by jointly intended coordination. Here we investigated whether bottlenose dolphins could understand a cooperative partner's role by testing whether they could learn a button-pressing task requiring precise behavioural synchronization. Specifically, members of cooperative dyads were required to swim across a lagoon and each press their own underwater button simultaneously (within a 1 s time window), whether sent together or with a delay between partners of 1–20 s. We found that dolphins were able to work together with extreme precision even when they had to wait for their partner, and that their coordination improved over the course of the study, with the time between button presses in the latter trials averaging 370 ms. These findings show that bottlenose dolphins can learn to understand their partner's role in a cooperative situation, and suggest that the behavioural synchronization evident in wild dolphins' synchronous movement and coordinated alliance displays may be a generalized cognitive ability that can also be used to solve novel cooperative tasks.

## 1. Introduction

Cooperation is found across the animal kingdom, from humans [1] to fishes [2] to baboons [3] to dolphins [4]. Cooperative behaviour in non-human animals can manifest in a number of ways, including cooperative breeding where all group members help raise young produced by the dominant breeding pair [5,6]; sexual coercion where males work together to either monopolize female groups [7] or contest access to individual females [8,9]; and cooperative hunting where individuals work together to secure large or difficult to catch prey items [10,11]. Studies have demonstrated, however, that animals can behave in ways that function cooperatively without actively and intentionally cooperating (e.g. [12,13]). In Brazil, for example, bottlenose dolphins drive fish towards human fisherman, a practice that the humans interpret as interspecies cooperation [14,15]. However, it may be that the fishermen simply act as an effective barrier, much like other barriers that dolphins herd fish against [16,17]. Successful cooperation such as this does not necessarily require an understanding of the cooperative role that others are playing [18]. To examine this question of understanding, experimental evidence is required.

To date, the cognitive mechanisms underlying animal cooperation have largely been explored in experiments using cooperative pulling tasks, in which two animals must simultaneously pull two ropes or handles in the same direction in order to receive a food reward [19]. The extent to which animals understand the cooperative nature of the task has been assessed by examining the following measures: (i) whether they pull more often if their partner is at the apparatus [13,20–23]; (ii) how often they glance at their partner during a cooperative situation [22,24–26]; (iii) whether blocking visual access between partners decreases success [22]; (iv) whether animals will wait to act until a delayed partner arrives [12,27–34]; and (v) whether they will actively recruit a partner [25,34]. These studies have suggested that chimpanzees [20,21,24,25,34], orangutans [26], capuchins [13,22], elephants [27], wolves [28], hyaenas [23] and keas [29] all take account of their partner to some extent, whereas otters [30], rooks [12], ravens [31] and the African grey parrot [32] may not. However, while actively recruiting a partner clearly demonstrates that an individual has an explicit understanding of their partner's role, as shown for chimpanzees [34], the extent to which these other assessment measures demonstrate such an understanding is not so clear. While waiting for a partner may indicate that animals understand their partner's role, the act of pulling (or pulling more) when a partner is present could be owing to response facilitation, in which one animal interacts with the apparatus because the other animal is doing so [35], or to an associatively learned rule that pulling is rewarded in the presence of a partner or of some environmental cue that the partner brings about (e.g. tension or movement in the rope) [36]. Similarly, glancing at the partner during cooperative tasks might theoretically be owing to monitoring for such learned environmental cues.

In general, for animals to succeed at these cooperative pulling tasks, it is necessary that individuals act during the same time period. However, it is not necessary that they precisely coordinate their behaviour. In experiments in which repeated solo pulling is not regulated, both animals may succeed by chance co-production if they repeatedly pull at the apparatus during the same time frame (e.g. [37]). In experiments in which solo pulling leads to a disabled mechanism (i.e. the ‘loose string’ paradigm), there is a small delay after one animal starts to pull before the rope is pulled out of the reach of the other. Animals may take advantage of this interval to act, as shown by the fact that otters performed better with a longer rope [30], which necessarily increased this delay and further relaxed the need for close synchronization. Thus, it may be that the less stringent timing requirements of these tasks create a window of opportunity for animals to perform successful behavioural strategies (e.g. ‘pull when a partner is there’ or ‘pull when the rope or tray starts moving’), even in the absence of jointly intended coordination.

Our goal in the current study was to investigate whether bottlenose dolphins could understand their partner's role in a cooperation task by testing whether they could learn a task that requires precise behavioural coordination between two partners. The bottlenose dolphin's propensity for cooperative behaviour makes them a model study subject for this type of task. For example, one of the most striking cases of cooperation in the animal kingdom is to be found in Shark Bay, Western Australia, where male Indo-Pacific bottlenose dolphins (*Tursiops aduncus*) are well known for their formation of nested alliances [38]. Pairs or trios of allied male dolphins cooperate together to herd single oestrous females [38], and these herding events can last for periods of less than 1 h to several weeks [38]. Multiple pairs or trios of males also cooperate in joint attacks on other alliances in order to steal females, or defend against such attacks [9]. In addition, these males exhibit highly synchronous and coordinated behaviour [39]. In other populations, common bottlenose dolphins (*Tursiops truncatus*) engage in cooperative feeding strategies requiring coordinated action. For example, a group of dolphins may rush simultaneously through shallow water onto the shore, creating a bow wave that strands fish in front of them to be easily grabbed [17,40], or one dolphin may drive a school of fish into a barrier of other dolphins [16] or human fishermen [14,15] waiting side by side. These cooperative feeding and reproductive partnerships may be central to each dolphin's survival and reproductive success [38], yet it remains unknown whether dolphins have an understanding of their partners' role during such cooperative interactions [41,42]. For this, experimental evidence is necessary.

Here, our task required members of cooperative dyads to swim across a lagoon and each press their own underwater button simultaneously (within a 1 s time window), whether sent together or with a delay between them of 1–20 s. The need for such tight behavioural synchronization eliminates the possibility that consistent success could be achieved from such mechanisms as response facilitation, following an associative rule to push the button in the partner's presence, or responding to some environmental cue brought about by the other's response.

In order to investigate how individuals modified their behaviour during these trials we measured a number of variables. First, while previous studies focused on whether the animal that reaches the apparatus first will wait for its partner, we hypothesized that the behaviour of the *delayed* animal may also provide useful information about the dyad's understanding of the task. Specifically, during short delay intervals, it is possible for the dyad to succeed if the delayed animal swims quickly to catch up to its partner. If both animals are actively coordinating, however, this is not necessary. We therefore predicted that individual swim speeds should decrease as dyads came to understand the cooperative nature of the task and thus increased their coordination. Second, we hypothesized that once individuals were synchronizing their button presses then the individual that arrived at the button first would not necessarily be the individual that pressed their button first. We therefore predicted that once the dyad understood the cooperative nature of the task, both the proportion of first button presses by the dolphin released first and the time between button presses should also decrease.

## 2. Methods

### (a) Subjects

Experiments were conducted at Dolphin Research Center (DRC) in Grassy Key, Florida between March 2017 and February 2018. The subjects were four common bottlenose dolphins: Gypsi (female, 10 years old) and Flagler (male, 6 years old) who formed dyad 1; and Aleta (female, 33 years old) and Calusa (female, 17 years old) who formed dyad 2. All four animals were born at DRC and were housed in natural seawater lagoons (ranging from 344 to 537 m^2^) with depth dependent on tide (4.5–5.5 m). The members of each dyad had lived together at various points throughout their lives, and lived together during the study. A third dolphin (Louie, male, age 7 years) also lived with Flagler and Gypsi during part of the study, but was gated into a different lagoon during experimental sessions.

All dolphins at DRC participate in three to five positive reinforcement training sessions daily, which may include husbandry, behavioural training, play sessions, public interactions with trainers and guests, and research. Behavioural training includes solo and tandem physical behaviours (e.g. asking two dolphins to dive together), as well as conceptual behaviours (e.g. repeat, imitate, do something new). Throughout the study, the dolphins were fed according to their normal daily routine, which typically included capelin, herring, smelt, and squid three to five times per day, approximately 20–33% of which they received during each experimental session (up to two sessions per day).

### (b) Cooperative task apparatus and procedure

**Figure 1.**
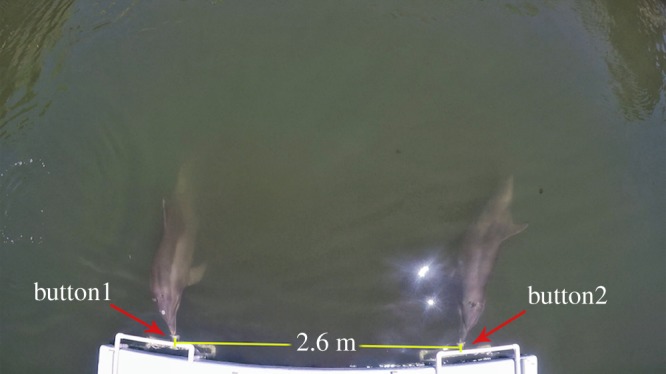
Aerial view of the cooperative task apparatus, with the buttons being pressed by one of the dolphin dyads.

The apparatus consisted of two underwater push buttons with pressure sensors (Hydracon Subsea Limit Switch) covered by 10 × 10 cm black starboard, mounted on 65 × 30 cm white starboard backed with neoprene (for sound dampening). The buttons were positioned off the centre of a dock, 53 cm below the water's surface and 2.6 m apart (measured from the centre of button 1 to the centre of button 2; see figure 1). The buttons were connected via a computer (Raspberry Pi Model 3 B+), which in turn was attached to an underwater speaker (University Sound UW-30). A researcher was positioned behind each button, hidden from the animals' view by a screen constructed of PVC pipe and dark cloth. Attached to each screen was a plastic tube through which the researchers could slide fish to positively reinforce the dolphins during successful trials. A Canon Vixia HF R50 video camera positioned across the lagoon from the apparatus and a GoPro Hero5 positioned above the apparatus were used to record the trials.

Each trial began with both dolphins and their respective trainers located at the opposite side of the lagoon (approx. 11 m away) from the task apparatus. During simultaneous release trials, the trainers each gave the ‘press the button’ hand signal simultaneously to their respective dolphins, at which point the dolphins were expected to swim across the lagoon and press their respective buttons simultaneously (within a 1 s window). If the buttons were pressed within this time interval, the computer automatically played a ‘success’ sound (i.e. a trainer's whistle) through the underwater speaker, and the researchers behind the screens slid fish through the feeding tubes to the dolphins. For dyad 2, the fish tubes were eliminated early in training (during phase 1; see table 1) owing to wintering seagulls and pelicans dive-bombing the fish that came out of the tubes. Instead, upon hearing the ‘success’ sound, these dolphins returned to the trainers across the lagoon for reinforcement. This procedure was followed for the remainder of the study. If there was more than a 1 s delay between the dolphins' button presses (or more than a 2 s delay during the initial preliminary training phase), the computer played a ‘failure’ sound, and no fish was given. The computer was programmed such that for each trial only the first press of each button was relevant, so it was impossible for dolphins to succeed by repeatedly pushing their buttons. The procedure for delayed-release trials was identical except that one dolphin was given the signal first (target animal), and the second dolphin was given the signal after a 1–20 s delay (delayed animal). The computer automatically recorded the following parameters for each trial: time between button presses (accurate to 0.01 s), which button was pressed first, and whether the trial outcome was a success or failure.

**Table 1.** Summary of all trial phases.

	trial type	criterion to pass
phase 0	simultaneous release	8 out of 10 over two sessions (80%)
phase 1	incremental delays (1–5 s)	3 in a row
phase 2	randomized delays (simultaneous – 5 s)	16 out of 20 in a single session (80%)
phase 3	incremental delays (8–20 s)	3 in a row
phase 4	randomized delays (1–20 s)	test (20 trials per dolphin)

### (c) Preliminary training

The experimental task was designed to capitalize on natural dolphin behaviour such as synchrony [39] and the use of their rostrums to manipulate objects and/or probe substrate [43]. However, the strict timing requirements coupled with the invisible causality of the task (e.g. no food that moved slowly towards them when they started the correct behaviour) meant that the means for success was not likely to be discovered spontaneously. We therefore implemented the following training steps: (i) from beside the buttons, each dolphin was taught to press a button when requested, by pairing the trainer's hand signal with a point towards the button and a target pole to guide them to the correct location; (ii) the target pole and trainer's point were faded out so that each dolphin pressed the button when given only the hand signal; (iii) the signals were given simultaneously to both dolphins, and the dolphins were required to press their buttons within a 2 s window of each other; (iv) the starting location was moved directly across the lagoon from the buttons, so that the dolphins had to swim across the lagoon to touch the buttons after being given the signals. Note that at this point the trainers initially pointed to the apparatus after giving the signal, before fading the point out again; and finally (v) the timing requirement was tightened so that the dolphins had to press the buttons within a 1 s window. By the end of this training phase, the dolphins were swimming together from the opposite side of the lagoon when given the signal, and pressing the buttons simultaneously (within a 1 s window). In line with previous studies [27], this tells us little about whether the animals understand the cooperative nature of the task, nor whether they are capable of coordinating their actions, but it does allow them to become familiar with the task itself. In order to move to the next stage the dyad had to succeed in 8 out of 10 trials on two consecutive days. Both dyads met this criterion.

### (d) Incremental delays

In the next phase we introduced increasing delays in which one of the dyad was asked to push the button before its partner. A trainer would use the hand signal to ask one member of the dyad to press the button, and then give no further instruction. A second trainer would wait for the predetermined delay duration before asking the second animal to press the button. To succeed, the first animal would need to wait for its partner and then precisely coordinate pushing their buttons. Owing to the fast nature of dolphins and the narrow time window required for success, delay durations were first increased in 1 s increments from 1 s up to 5 s to allow them to learn the task, and then in 3 s increments between 8 and 20 s. For dyad 1, we originally attempted to increase the intervals in larger increments, moving directly from 0 to 2 to 5 s intervals. However, the dolphins had difficulty with the 5 s delay and began to show behavioural indications of frustration. Therefore, we started again with simultaneous trials and 2 s delays, then moved in 1 s increments until they had passed 5 s. For dyad 2, we used 1 s increments for 1 to 5 s delays from the beginning. During this period, members of the dyad alternated being the target dolphin (i.e. the one who was released first). If the dyad was unsuccessful for three trials in a row with one dolphin as target, the other dolphin would become the target. If a target dolphin successfully waited for its partner three trials in a row it passed that delay duration. When the first of the dyad passed a delay interval we continued to test its partner at that duration. If that partner was unsuccessful three trials in a row the target temporarily switched back to the dolphin who had already passed for two trials (regardless of outcome). This ensured that the individual who had mastered the task remained engaged. The maximum number of trials in a session was 20, with no more than two sessions per day.

### (e) Randomized delays

In theory, learning the required amount of waiting time or required swimming speed could solve predictable delays. To make sure the dolphins could not use such strategies, we randomized the delays at two points: (i) after the dyad succeeded at a 5 s delay we presented them with randomized trials of 0, 2, 3, 4 and 5 s delays until the dyad succeeded in 16 out of 20 trials (80% success rate) in a given session; and (ii) after the dyad succeeded at a 20 s delay they were tested with randomized delay trials from 1 through to 20 s. This consisted of one trial for each target dolphin at every possible delay between 1 and 20 s, for a total of 40 trials per dyad tested over three sessions. Both the order of the delay intervals and the target animal were randomized, with the constraint that one individual could not be the target dolphin for more than three consecutive trials. These randomized trials allowed us to ensure that animals were not passing the task by becoming familiar with successive delays, but understood that irrespective of the delay time they needed to wait for their partner. A full summary of trial phases for each dyad is presented in table 1.

### (f) Analysis

All statistical procedures were conducted in R v. 3.3.2 [44]. To determine whether and how success strategies evolved over the course of the study we ran mixed-effect models (lmer and glmer using *lme4* package in R) on a number of behavioural parameters for successful trials: (i) we used the event logging software BORIS [45] to code the time it took for the delayed animal to swim across the lagoon for successful trials. This was measured from when the trainer gave the delayed animal the ‘press the button’ hand signal to the ‘success’ sound being played. To explore how the delayed animal modified their swim speed across the study we ran a linear mixed model (LMM) on the swim time of the delayed animal. The model predictor was trial phase, which was modelled as four distinct phases (phases 1–4, table 1). To control for repeated measures of individuals, the identity (ID) of the delayed individual was included as a random effect. The full model was compared to a null model containing only the random effects, and we selected the model with the lowest Akaike's information criterion (AIC) value as the best-fitting model. We also employed ANOVA using the *car* package in R to test whether the inclusion of the trial phase parameter in the model explained significantly more variance; (ii) to explore how cooperative timing changed across trials for the animal being tested we ran a generalized linear mixed model (GLMM) with binomial family on the number of successful trials where the target animal pressed their button first, i.e. before their delayed partner (1 = yes, 0 = no), with trial phase as an explanatory factor variable and target individual ID included as a random effect. Model selection was as per previous analysis; (iii) finally, we ran a LMM on the time between both individuals pressing their button for all successful trials. Trial phase was included as an explanatory factor variable and target individual ID was included as a random effect. Model selection was as per previous analysis.

## 3. Results

**Figure 2.**
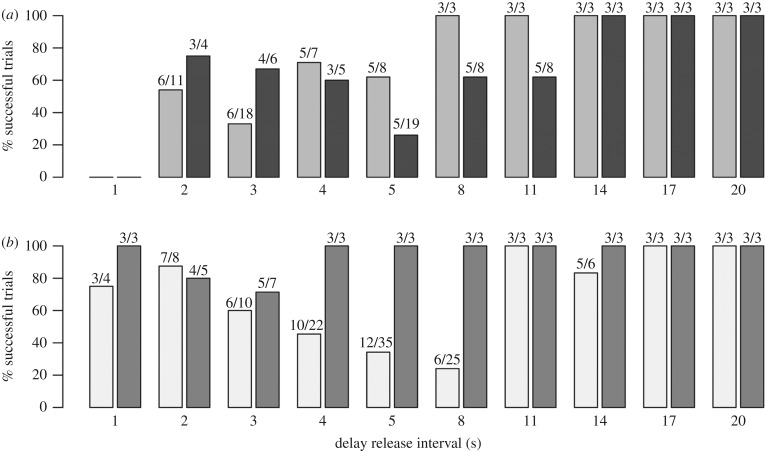
Summary of the percentage of successful trials for each individual across all delay release intervals; where an individual had to pass three trials in a row in order to move to the next interval. The number of successful trials over the total number of trials attempted is also provided per interval. Panel (*a*) shows results for dyad 1 (Flagler in light grey, Gypsi in dark grey) and panel (*b*) shows results for dyad 2 (Aleta in white, Calusa in grey). Note, one second delays were not tested for dyad 1.

**Figure 3.**
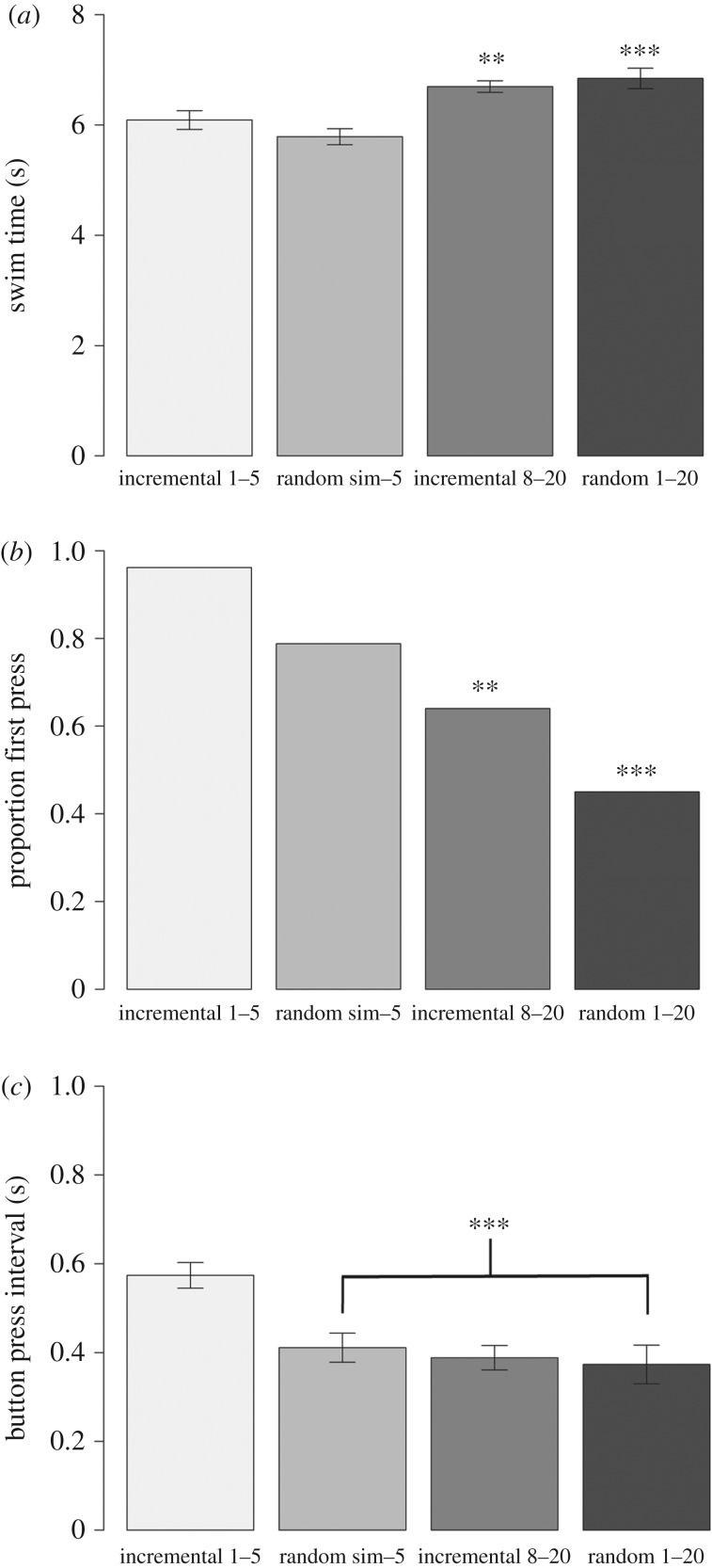
Summary of behavioural strategies for all successful trials across all individuals and trial phases: (*a*) swim time of delayed animal; (*b*) proportion of successful trials in which the target dolphin pressed their own button before their partner pressed; and (*c*) averaged elapsed time between the button presses. The asterisks indicate a significant difference (**p ≤ 0.01; ***p < 0.001).

After reaching the criterion of at least 8 out of 10 successful simultaneous release trials over two days, both dyads successfully passed the incremental delay release trials with the delay ranging from 1 to 20 s (figure 2). However, there was variation between individuals in how quickly they learnt that they needed to wait for their partner in order to successfully complete the task. In both dyads it appears that one individual had a faster learning rate compared to its partner (Flagler in figure 2*a*, Calusa in figure 2*b*). Interestingly, in the earlier successful trials the delayed animal swam significantly faster (i.e. had shorter swim times) than in the later successful trials (phase 1 versus phase 3, lmer: *t* = 2.9, *p* = 0.003; and phase 1 versus phase 4, lmer: *t* = 7.2, *p* < 0.0001; figure 3*a*, table 2), suggesting that initial strategies focused on the delayed animal ‘catching up’ rather than the target animal waiting. The proportion of first button presses by the target animal also significantly decreased over the course of the trials (phase 1 versus phase 3, glmer: *z* = −3.13, *p* = 0.001; and phase 1 versus phase 4, glmer: *z* = −4.07, *p* < 0.0001; table 2), as did the time between button presses (phase 1 versus phase 2, lmer: *t* = −5.2, *p* < 0.0001; phase 1 versus phase 3, lmer: *t* = −4.9, *p* < 0.0001; and phase 1 versus phase 4, lmer: *t* = −5.2, *p* < 0.0001; table 2), indicating that individuals became better at coordinating their behaviour (figure 3*b*,*c*). Full model outputs are provided in the electronic supplementary material. Finally, all four animals were highly successful at the randomized delay trials (table 3), revealing their understanding of the cooperative nature of the task (example movies are available from the Dryad Digital Repository: http://dx.doi.org/10.5061/dryad.1pf43rb [46]).

**Table 2.** Summary results of the mixed models: effects of trial phase on swim speed, the proportion of first button presses by the individual released, and first time between both individuals pressing their button. (Phase 1 was the reference category.)

model	parameter	estimate	confidence interval
(LMM) swim speed of delayed animal	phase 2	−0.226	−0.494 to 0.044
	phase 3	0.350	0.114 to 0.586
	phase 4	1.079	0.788 to 1.370
(GLMM) first button press	phase 2	−0.522	−1.265 to 0.209
	phase 3	−0.996	−1.643 to −0.388
	phase 4	−1.454	−2.173 to −0.766
(LMM) button press interval	phase 2	−0.188	−0.259 to −0.118
	phase 3	−0.159	−0.224 to −0.096
	phase 4	−0.205	−0.282 to −0.127

**Table 3.** Success rates of individuals for the randomized delay trials.

target dolphin	delayed dolphin	no. successful trials	% successful
Gypsi	Flagler	19 out of 20	95%
Flagler	Gypsi	20 out of 20	100%
Calusa	Aleta	19 out of 20	95%
Aleta	Calusa	18 out of 20	90%

## 4. Discussion

In the current study, bottlenose dolphins (*Tursiops truncatus*) demonstrated an understanding of their partner's role in a cooperation task by waiting for their partner as in previous studies [12,27–34] and by precisely coordinating their behaviour in order to ensure task success. Furthermore, their behavioural strategies and the coordination between individuals significantly improved in accordance with their understanding of the task itself. During earlier trials, delayed individuals swam significantly faster, and the time between partners' button presses was significantly longer, suggesting that initial strategies focused on the delayed animal catching up to its partner rather than the target animal waiting and the partners precisely coordinating their behaviour. Such coordinated behaviour was evident by phases 3 and 4 where longer delays were introduced, with all four animals achieving high levels of task success once delayed intervals reached greater than 10 s. Behavioural coordination was evident by the combination of both significantly slower swim speeds and shorter times between button presses, conceivably once both members of the dyad understood that rapid swimming was not required for task success, rather that jointly coordinated action was.

One might question whether the dolphins' success could be explained by a simpler individual behavioural strategy rather than by jointly coordinated action. Note that in phase 4 the average timing difference between partners' button presses was 370 ms. This level of precision makes it virtually impossible that the dolphins were reacting to some general cue such as ‘press when a partner is near the apparatus’, and highly unlikely that they were responding to a more specific perceptual cue that their partner had pressed the button. Moreover, if one partner had been initiating their button presses on the basis of such a perceptual cue, then the data would show that one partner of the dyad (the reacting partner) consistently pressed their button after the other partner (the cueing partner) did. This is not what happened. Instead, by phase 4, the trials of all of the target dolphins were split between those in which they or their partner pressed first (figure 3*b*). This suggests that once the target animal understands its partner's role then it works with its partner to press the buttons simultaneously. Under this scenario we would expect the target animal to press their button first by chance, i.e. in approximately 50% of trials. As such, we have shown that bottlenose dolphins can precisely coordinate their behaviour in a cooperative task.

It is perhaps worth noting that the dolphins in the current study were required to learn that this was a cooperative task by trial and error. Unlike in previous cooperation studies, the task here included no perceptible causality by which they might deduce that they must work together (and indeed, during the initial introduction to the apparatus, pushing a button solo resulted in success). While this rendered the task more challenging than in previous studies, it also meant that shorter intervals, and therefore more extensive training, were required in the initial stages. It still remains to be seen whether dolphins might immediately employ a similar collaborative strategy on a cooperation task that is more causally transparent.

Nonetheless, the current study has provided evidence that dolphins are capable of joint action, defined as the ability to coordinate actions with others in order to reach common goals [47]. In wild dolphins, synchrony occurs in a variety of contexts, such as synchronous breathing between mothers and calves [48], and behavioural synchrony between allied males in coordinated displays [39,49]. Indeed, motor synchrony between allied male dolphins is remarkably precise, with synchronous behaviours separated by just 130 ms [39]. Such synchrony is thought to promote both coordination and cooperation between alliance partners [50]. Our results, therefore, suggest that the tight behavioural coordination which bottlenose dolphins show in the wild may be a generalized cognitive ability that they can also apply to novel, albeit artificially constructed, cooperative situations. Future studies should explore whether other species can also pass cooperative tasks that require precision in motor synchrony and, thus, are capable of joint action.

Finally, in previous studies, chimpanzees' ability to actively recruit a partner demonstrated that they have a clear understanding of their partner's role in a cooperative context [34]. It has been proposed that the competition inherent in chimpanzee societies has favoured complex cognitive mechanisms underlying cooperation [36], allowing individuals to actively compete over the best cooperative partners [51]. Partner choice also plays a central role in some bottlenose dolphin populations where males form multi-level alliances as a means of enhancing reproductive success [38]. Such strategic behaviour can place a demand on higher cognitive abilities, particularly for species where mobility leads to encounters with many potential cooperative partners [52]. Here we have shown that bottlenose dolphins are capable of precise joint action during a cooperative task, supporting the notion that in species where biological markets are prevalent [51], individuals appear to possess the cognitive skills that enable them to know enough about their partner to use them as social tools [36]. Future studies should test whether dolphins, like chimpanzees, are also capable of partner recruitment during novel cooperative tasks, thus demonstrating an even more complete and flexible understanding of the partner's necessity and role in cooperative situations.

## Supplementary Material

Jaakkola et al. ESM

## Supplementary Material

Cooperation data file for ProcB

## Data accessibility

The datasets supporting this article have been uploaded as part of the electronic supplementary material. All data are archived and accessible at the Dolphin Research Center. Example movies are available from the Dryad Digital Repository: http://dx.doi.org/10.5061/dryad.1pf43rb [46].
