# Supplementary material for: Bottlenose dolphins can understand their partner's role in a cooperative task
Source: Proc Biol Sci. 2018 Sep 19;285(1887):20180948. doi: 10.1098/rspb.2018.0948 (PMC6170804; doi:10.1098/rspb.2018.0948)
Supplement: Jaakkola et al. ESM [file rspb20180948supp1.docx]

**Supplementary Material**

**Bottlenose dolphins can understand their partner’s role in a cooperative task**

Kelly Jaakkola^1*^, Emily Guarino^1^, Katy Donegan^1^, Stephanie. L. King^2*^

^1^ Dolphin Research Center, 58901 Overseas Highway, Grassy Key, FL 33050, USA

^2^ Centre for Evolutionary Biology, School of Biological Sciences, University of Western Australia, Crawley 6009, Australia.

*Corresponding authors: [kelly@dolphins.org](mailto:Kelly@dolphins.org) and [stephanie.king@uwa.edu.au](mailto:stephanie.king@uwa.edu.au)

All data used in the models are provided in the electronic supplementary material (Cooperation data file for ProcB Excel file)

**Table S1. (a)** Model selection for the linear mixed model results for swim speed of the delayed animal. Visual inspection of the model residuals revealed nine outliers (swim speeds > 10 seconds). These were removed from the model to achieve normality of model residuals (confirmed with diagnostic plots). The removal of these outliers had no significant effect on effect size, direction of effect or p values.

|  | Model | AIC | Δ AIC | Pr(>Chisq) |
| --- | --- | --- | --- | --- |
| Full | lmer(delayed swim ~ factor(trial phase)+(1\| Delayed individual ID)) | 1304 |  | <0.0001 |
| Null | lmer(delayed swim ~ 1+(1\| Delayed individual ID)) | 1368 | 64 |  |

**(b)** Parameter estimates for a linear mixed model predicting swim speed of the delayed animal as a function of trial phase (1-4).

| Estimate Std. Error df t value Pr(>\|t\|) |
| --- |
| Intercept 6.258 0.642 4 9.750 0.00053 ***  Phase 2 -0.226 0.138 447 -1.642 0.10134  Phase 3 0.350 0.121 447 2.902 0.00389 **  Phase 3 1.079 0.149 447 7.258 < 0.0001 *** |

**Table S2. (a)** Model selection for the generalised linear mixed model results for proportion of successful trials where the target dolphin pushes their button first. Diagnostic plot revealed no major departure from model assumptions using method for generalized linear mixed effect models described in [1]

|  | Model | AIC | Δ AIC | Pr(>Chisq) |
| --- | --- | --- | --- | --- |
| Full | glmer(button press first ~ factor(trial phase)+(1\| Target individual ID),family=binomial) | 498 |  | 0.0001 *** |
| Null | glmer(button press first ~ 1+(1\| Target individual ID)) | 513 | 15 |  |

**(b)** Parameter estimates for a generalised linear mixed model with binomial family predicting proportion of successful trials where the target dolphin pushes their button first as a function of trial phase (1-4).

| Estimate Std. Error z value Pr(>\|z\|) |
| --- |
| Intercept 1.652 0.329 5.022 < 0.0001***  Phase 2 -0.522 0.373 -1.399 0.16194  Phase 3 -0.996 0.318 -3.132 0.00174 **  Phase 4 -1.454 0.357 -4.072 < 0.0001*** |

**Table S3. (a)** Model selection for the linear mixed model results for elapsed time between button presses. Diagnostic plots confirmed normality of model residuals.

|  | Model | AIC | Δ AIC | Pr(>Chisq) |
| --- | --- | --- | --- | --- |
| Full | lmer(button press time ~ factor(trial phase)+(1\| Target individual ID)) | 111 |  | <0.0001 |
| Null | lmer(button press time ~ 1+(1\| Target individual ID)) | 129 | 18 |  |

**(b)** Parameter estimates for a linear mixed model predicting elapsed time between button presses as a function of trial phase (1-4).

| Estimate Std. Error df t value Pr(>\|t\|) |
| --- |
| (Intercept) 0.530 0.029 19 17.98 < 0.0001 ***  Phase 2 -0.189 0.036 419 -5.202 < 0.0001 ***  Phase 3 -0.159 0.032 459 -4.919 < 0.0001 ***  Phase 4 -0.205 0.039 459 -5.195 < 0.0001 *** |

**References**

1. Zuur AF, Saveliev AA, Ieno EN, Smith GM, Walker N. 2009 *Mixed Effects Models and Extensions in Ecology with R*. New York: Springer-Verlag.
